# Impact of creatine supplementation and exercise training in older adults: a systematic review and meta-analysis

**DOI:** 10.1186/s11556-025-00384-9

**Published:** 2025-10-08

**Authors:** Ghazal Sharifian, Parastou Aseminia, Diako Heidary, Joseph I. Esformes

**Affiliations:** 1 Faculty of Pharmacy, Tehran University of Medical Sciences, Tehran, Iran, Islamic Republic of; 2 Faculty of Physical Education and Sport Sciences, Allameh Tabataba’i University, Tehran, Iran, Islamic Republic of; 3 Cardiff School of Sport and Health Sciences, Cardiff Metropolitan University, Cardiff, UK

**Keywords:** Aerobic exercise, Resistance training, Older adults, Creatine supplementation

## Abstract

The aim of this study is to assess the effects of the combination of and exercise training on older adults’ physical performance and body composition. This meta-analysis was conducted according to the Preferred Reporting Items for Systematic Reviews and Meta-Analyses (PRISMA) guidelines. Scopus, Web of Science, and PubMed databases (date of access: 30 August 2024) were queried. Twenty articles met the inclusion criteria and a total of 1093 participants (69% females and 31% males) were included in the study. The mean difference (MD) with 95% confidence intervals (CIs) and the overall effect size was calculated for all comparisons between the creatine plus exercise training group and placebo plus exercise training group. The PEDro scale was used to evaluate the quality of articles. Our findings showed that creatine intake and exercise training significantly affect 1RM (kg) in older adults (mean difference = 2.122, Z = 3.255, *P* = 0.001). There was a significant effect on fat percentage (%) in older adults (mean difference = -0.548, Z = -2.231, *P* = 0.026), while creatine intake and exercise training did not have a significant effect on bone mineral density (BMD) of total body (g/cm^2^) in older adults (mean difference = 0.009, Z = 0.587, *P* = 0.557). By participating in exercise training programs and creatine supplementation, older adults can improve their functional performance and body composition. However, the effects of exercise training and creatine supplementation on BMD require further study (PROSPERO registration number: CRD42024581817).

## Introduction

The World Health Organization (WHO) defines aging as the accumulation of bodily damage, leading to a gradual physical and mental health decline over time [1]. From 2000 to 2021, the global average healthy life expectancy at birth increased by 3.57, reaching 61.9 years [2]. The latest WHO report on the world’s older adult population estimates that by 2050, the proportion of adults aged 60 years and older will nearly double to 22% [1]. As birth rates continue to decline worldwide, the population’s age distribution is shifting toward a greater proportion of older individuals [3].

As people age, they often face a range of physical and mental health challenges known as geriatric syndromes, including cardiovascular diseases, mobility impairments, and cognitive dysfunction. However, many of these problems can be prevented or improved by adopting a more active lifestyle [4–6]. A lack of physical activity and exercise is directly linked to health issues such as hypertension, coronary heart disease, osteoporosis, and diabetes mellitus [7, 8].

Creatine is an ergogenic substance that enhances physical performance [9],and it has been extensively studied and used since its discovery in 1832, particularly among athletes [10, 11]. Also known as methylguanidine-acetic acid, creatine is naturally produced through a series of reactions involving the amino acids arginine, glycine, and methionine, primarily in the liver and kidneys [12]. Creatine supplementation has physiological benefits in older adults, primarily enhancing muscle strength, lean mass, and functional capacity [13]. It supports cellular energy metabolism by replenishing ATP stores, enhancing mitochondrial efficiency, and reducing fatigue during physical exertion [14, 15]. These combined effects emphasize creatine’s role in reducing age-related muscle and bone health decline while enhancing overall physical function [16].

Several studies indicate that creatine can have therapeutic effects on various clinical conditions when taken as a nutritional supplement [17]. However, without exercise support, creatine alone does not improve muscle strength, physical performance, or overall physical and mental health [18]. Many studies have assessed the impact of various supplements on physical performance, with mixed results [19]. Due to conflicting research findings, the effectiveness of creatine on physical and mental health in older adults remains debated [19]. For example, Brose et al. conducted a study on older adults that examined the impact of creatine supplementation on isometric strength and body composition following strength training [20], showing enhanced muscle strength and improved performance in functional tasks. In contrast, Alves et al. found no significant effects of creatine on strength training, cognitive function, or emotional parameters [21].

Several systematic reviews and meta-analyses have previously highlighted the impact of creatine monohydrate (CrM) supplementation combined with exercise training in aging adults (48 years old and above) [22–24]. Earlier reviews, such as those by Candow et al. (2024 and 2025) [14, 23], summarized general findings regarding CrM supplementation benefits on muscle and bone health in older adults, indicating its potential to enhance muscle gain and manage osteosarcopenia. Stares et al. [24] also reviewed and summarized the additive ergogenic effects of creatine on muscles when combined with resistance training and suggested a prolonged duration for improving bone density. Similarly, Devries and Phillips (2014) [22] meta-analysed the effects of CrM with resistance training (RT) in older adults, concluding that CrM + RT improved total body mass, fat-free mass, as well as upper and lower body strength. More recently, dos Santos et al. [25] provided a meta-analysis focused on older females (≥ 60 years), reporting that CrM + RT enhanced muscle strength.

While comprehensive, these prior works are constrained by earlier literature search dates, with some relying on qualitative syntheses, or limited to singular sex or specific types of exercise training. Thus, an updated quantitative analysis is needed to address lingering questions and account for the continuously evolving body of research. The current meta-analysis addresses these gaps by incorporating the most recent randomized clinical trials (literature search extended to August 2024) into a rigorous quantitative analysis to examine the additive effects of creatine supplementation during various types of exercise training on physical performance, body composition, and bone mineral density in older adults aged 55 years and above.

## Methods

### Literature review and search strategy

The study protocol for this systematic review has been submitted to the International Prospective Register of Systematic Reviews (PROSPERO) database (registration number: CRD42024581817) and follows the guidelines set forth by the Preferred Reporting Items for Systematic Reviews and Meta-Analyses (PRISMA) [26]. The Scopus, Web of Science, and PubMed databases were accessed on 30 August 2024 using the following search terms: (“exercise training” OR “aerobic exercise” OR “aerobic training” OR “resistance training” OR “resistance exercise” OR “physical activity”) AND (“creatine” OR “creatine supplementation” OR “creatine supplement”) AND (“older adults” OR “elderly” OR “aging” OR “aged” OR “elders”). These three databases were chosen for their credibility in meta-analyses and their comprehensive, up-to-date indexing of peer-reviewed publications across various disciplines [27]. The risk of selection bias was mitigated by employing multiple databases with various indexing practices, broad search terms and manual screening of the reference list entries to ensure the coverage of all pertinent literature.

No restrictions were placed on the year of publication for the studies included. The selected studies comprised randomized controlled trials (RCTs) that involved an exercise intervention, creatine supplementation, and participants aged 55 years and older. No restrictions were placed on the year of publication for the studies included. We defined individuals aged 55 and older as representative of the older adult population because the prevalence of chronic conditions linked to aging, such as hypertension, obesity, and arthritis, begins to rise at this age. These conditions often double or triple in frequency as individuals age [4, 28, 29]. Furthermore, approximately one-fifth of individuals within this age group experience difficulty with physical activities such as walking and bending, which is associated with the observed decline in physical activity that commences at this stage of life [28–30]. We excluded studies that had exercise interventions lasting less than two weeks, used mixed creatine formulations (i.e., combined with protein or other dietary supplements), or involved animal subjects. For all included studies, the comparator group was defined as participants receiving placebo supplementation combined with the same exercise training plan as the intervention group. The outcome measures selected for meta-analysis were one-repetition maximum (1RM), fat percentage, and bone mineral density (BMD). These outcomes were chosen due to their clinical relevance in assessing age-related physiological changes and their consistent reporting across a sufficient number of included studies, allowing for robust quantitative synthesis.

### Selection process

The search results were exported to the EndNote X8 citation manager, where duplicates were removed. Two reviewers independently screened the titles and abstracts for eligibility based on the inclusion and exclusion criteria, and in cases of disagreement, a third reviewer determined the final action. They then checked the full texts of the remaining articles against the inclusion criteria. Each article was assigned a score: 1 for inclusion and 0 for exclusion. In cases where there were discrepancies between the reviewers’ scores, the final decision was made based on the opinion of a third reviewer.

### Quality and risk of bias assessment

The quality of the selected studies was evaluated using the Physiotherapy Evidence Database (PEDro) scale, which consists of 11 scoring items based on the *Delphi* list to assess the methodological quality of RCTs [31, 32]. The maximum score on the scale is 10 because the first criterion, which represents the applicability of the trials, is not included in the total score calculation [31]. A score below 4 on the PEDro scale indicates “poor” methodological quality, while scores above 7 represent “high” methodological quality [33–35]. Two reviewers independently evaluated the studies, awarding points based on clear satisfaction with each item. Before finalizing the scores, the reviewers discussed any contradictions until a consensus was reached. Risk of bias for the included studies was evaluated with version 2 of the Cochrane risk-of-bias 2 tool for randomized trials (RoB 2) [36] by two independent reviewers. This tool assesses RCTs bias in five domains including the randomization process, deviations from intended interventions, missing outcome data, measurement of the outcome, and selection of the reported result. According to the RoB 2 tool scoring system, studies are categorized as having low risk of bias, some concerns, or high risk of bias.

### Data extraction

Reviewers extracted data from the selected articles. The extracted information included: (1) Authors and publication date; (2) Number of study participants; (3) Study location and target population; (4) Interventions, specifically the type of physical exercise and dosage of creatine supplementation; (5) Duration of the intervention; (6) Number of exercise sessions per week; (7) Summary of the findings. Also, numerical data related to the mean, standard deviation, and number of participants in the pre-test and post-test of the factors analyzed in the meta-analysis were extracted and recorded in a separate Excel file and then analyzed in the Comprehensive Meta-Analysis (CMA) software.

### Data synthesis and Meta-analysis

Statistical analyses were conducted using CMA Software Version 2.0. We performed a meta-analysis to assess changes in fat percentage, bone mineral density (BMD), and one-repetition maximum (1RM) data from pre-test to post-test by calculating the mean difference (MD) between the intervention and control groups, along with a 95% confidence interval (CI). The software was also used to evaluate heterogeneity among the studies (I²), and we employed Begg and Egger’s tests to detect any publication bias. To determine the correlation between pre-test and post-test results, we used an average value of 0.5, while the sensitivity analysis was assessed using the one-study-removed method.

## Results

### Literature search and screening

**Fig. 1:**
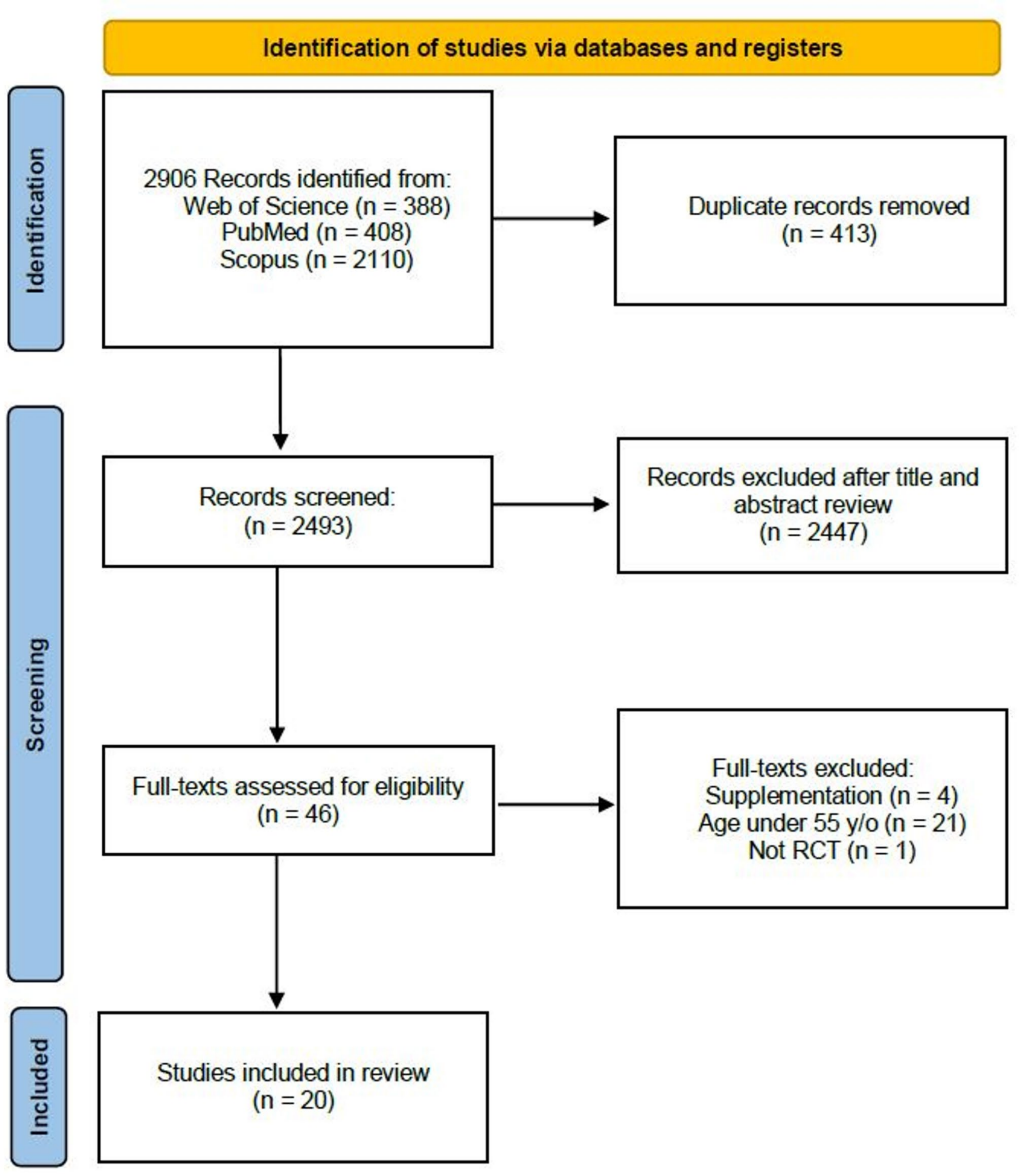
PRISMA workflow and search strategy flowchart

Two thousand nine hundred six references were exported after the initial database search (see Fig. 1). After removing duplicates and reviewing the titles and abstracts, 48 records remained. All publications selected for full-text review were written in English. Upon reviewing the full texts, 21 studies did not meet the population age criterion, 4 did not meet the supplementation criterion, and 1 was not an RCT. Ultimately, 20 studies were included for meta-analysis. The PEDro scale was used to assess the methodological quality of these studies. None of the studies scored below 4 on the PEDro scale (Table 1). Therefore, no studies were excluded from the meta-analysis based on their PEDro scores. The average PEDro score for the included studies was 7.95 ± 0.89. According to RoB 2 tool scoring results, eleven studies were considered as high risk of bias; six because of the effects of adherence to intervention and five due to the number of drop outs from the study and the vague explanation for their withdrawal. Five RCTs were marked with some concerns with four lacking information on a prespecified analysis plan and one with some concerns about the randomiztation process. Details of the study characteristics are reported in Table 1.

**Table 1:** Specifications of the included randomized controlled trials

No.	Study	Total sample size/Gender	Population	Intervention	Duration (week)	Session per week	Results	PEDro score	ROB2 score
1	Chilibeck et al. 2005 [37]	29/ Male: 29, Female: 0	Healthy older adults (70.8 ± 6.6 y)/Canada	Resistance training (RT) + 0.3 g/kg creatine for 5 d and 0.07 g/kg afterward	12	3	A 12-week RT program enhanced bone mineral density in older people, with possible added effects on regional bone mineral content if combined with creatine supplementation. The increase in bone mineral content can be attributed to the elevated tension at muscle attachment sites to the bones due to the creatine-enhancing effects on muscle mass.	7	H
2	Hass et al. 2007 [38]	20/ Male:17, Female:3	Patients with Parkinson’s Disease (PD) were ambulatory, clinically stable, non-fluctuating, and classified as Hoehn and Yahr stage 3 or lower in the United States.	20 g/d for the first 5 days and 5 g/d thereafter of creatine. Both groups participated in progressive RT (24 sessions, 2 Times per week, 1 set of 8–12 repetitions, 9 exercises).	12	2	Creatine monohydrate enhanced the exercise-induced gains in 1RM strength for the chest press and biceps curl and improved chair rise performance. Patients with PD Strength increased by 9% to 23% after 12 weeks of training, with additional benefits from creatine supplementation ranging from 13% for leg extensions to 188% for biceps curls.	7	S
3	Gualano et al. 2014 [15]	60/Male:0, Female: 60	Postmenopausal vulnerable women (with osteopenia or osteoporosis,$\:age\ge\:60\:$)/Brazil	RT + 20 g/day creatine for 5 days and 5 g/day afterward	24	2	In vulnerable older women, long-term creatine supplementation enhanced appendicular lean mass, and when combined with RT, it improved muscle function. The interventions did not significantly affect bone mass.	7	H
4	Chilibeck et al. 2023 [39]	237/ Male:0, Female: 237	Postmenopausal women (59.0 ± 5.6 y)/Canada	RT + walking + 0.14 (g/kg) creatine monohydrate for 2 years	104	RT:3 walking: 6	Creatine supplementation and exercise had no significant effect on bone marrow density but improved specific geometric properties of the proximal femur in postmenopausal women after two years.	9	L
5	Faager et al. 2006 [40]	23/Male:10, Female:13	Older chronic obstructive pulmonary disease (COPD) patients (66 ± 6 years) Sweden	A rehabilitation program, including exercise training + 0.3 g/kg/day creatine for 7 days and 0.07 g/kg/day afterward	8	2	The Endurance Shuttle Walking Test (ESWT) results showed that coupling oral creatine supplementation with eight weeks of exercise training had no advantage over exercise training alone in improving the physical performance of COPD patients.	8	L
6	Alves et al. 2013 [21]	56/Male:0 Female: 56	Older women (aged between 60 to 80 y)/Brazil	20 g/d creatine monohydrate for 5 d and 5 g/d afterward	24	2	A 24-week RT program improved emotional well-being and increased muscle strength in older women without affecting cognitive function. Creatine supplementation did not enhance the benefits of strength training or significantly influence cognitive function or emotional well-being.	7	H
7	Chrusch et al. 2001[41]	30/ Male: 30, Female: 0	Older men (70.4 ± 1.6 y)/Canada	RT + 0.3 g/kg creatine for 5 d and 0.07 g/kg afterward	12	3	In untrained older men, creatine supplementation enhances the effects of RT on muscle performance and the development of lean tissue. These benefits are even more significant in trained older men.	8	H
8	Oliveira et al. 2020 [18]	32/ Male: 11, Female: 16	Non-athletic older adults (aged between 60 to 80 y)/Canada	RT + 5 g/d creatine monohydrate	12	3	Twelve weeks of RT, regardless of its coupling with creatine supplementation, did not lower insulin resistance or inflammation markers, except for monocyte chemoattractant protein-1.	7	H
9	Brose et al. 2003 [20]	28/ Male: 15 Female: 13	Healthy men and women ($\:age\ge\:65\:$)/Canada	RT + 5 g/d creatine monohydrate	14	3	Fourteen weeks of RT enhanced muscle strength and improved functional task performance in the older population. Creatine supplementation further amplified the effects of RT on fat and bone-free mass, total body mass, and isometric knee extension strength.	8	H
10	Johannsmeyer et al. 2016 [42]	31/ Male: 17, Female: 14	Untrained aging adults (Age 58.0 ± 3 y and 57.6 ± 5 y)/Canada	RT + 0.1 g/kg creatine	12	3	Creatine supplementation enhanced muscle mass gains in a 12-week drop-set RT program for untrained aging adults, with more pronounced effects in males than females.	9	H
11	S. Bermon et al. 1998 [43]	32/Male:16, Female: 16	Healthy sedentary to moderately active (Age 67–80 y)/France	Strength training. 3 sets of leg press, knee extension, and chest press 8 reps. (5 g creatine monohydrate + 2 g glucose) 4 times/d for 5 d then (3 g creatine monohydrate + 2 g glucose) once daily in 8 males and 8 females.	7	3	All training groups demonstrated an increase in all movements, while non-training groups showed no significant results. The Creatine + training group exhibited greater effects than the placebo + training group. However, the overall results did not meet expectations.	7	S
12	Bert O. Eijnde et al. 2003 [44]	46/ all male	Healthy adults (age: 55–75 y)/Denmark	Cardiorespiratory endurance training and moderate RT. 5 g creatine monohydrate/day.	phase 1: 12 weeks (*n* = 46) phase 2: 24 weeks (*n* = 26)	2–3	After six months of training, the placebo and creatinine groups experienced increased leg extension and arm curl workloads. However, in month 12, compared to baseline, the leg extension workload increased while the arm curl workload decreased.	9	H
13	Rogers et al. 2006 [45]	44/ male: 21 female: 23	Healthy adults (age: 55–84)/United States	Strength training, 3 sets 8–12 reps. 3 g creatine/day	12	3	The RT program showed improvements in strength and lean mass, particularly in the creatine group, compared to the placebo, although only the bench press results were statistically significant.	7	S
14	Pinto et al. 2016 [46]	32	Healthy non-athlete (age: 60–80)/Brazil	RT + Creatine 5 g/d	12	3	Twelve weeks of low-dose creatine supplementation and resistance training increased lean mass among older adults.	8	H
15	Aguiar et al. 2013 [47]	18/ all female	Healthy non-athlete women (age: 64.9 ± 5 y$\:)$/Brazil	5 g/d creatine + RT 12 sets 10–15 reps	12	3	Long-term creatine supplementation, when combined with RT, enhances the capacity to perform submaximal strength functional tasks and increases maximal strength, fat-free mass, and muscle mass in older women.	9	S
16	D.G. Candow et al. 2008 [48]	35/all male	Healthy men (age: 59–77 y)/Canada	RT 3 sets of 10 reps + 0.1 g/Kg creatine on training days	10	3	The creatine group experienced a greater body mass and total thickness increase than the placebo group. Additionally, low-dose creatine reduced muscle protein degradation and bone resorption without increasing formaldehyde production.	8	H
17	Roschel et al. 2021 [49]	200/ male: 46 female: 154	Pre-frail or frail adults (age 72 ± 6 y)/Brazil	RT, load progressed every 4 weeks + 3 g creatine twice daily	16	2	Creatine supplementation did not improve RT adaptations in pre-frail and frail elderly individuals, regardless of sex.	9	L
18	Amiri et al. 2023 [50]	45	Non-athlete (age: 68.1 ± 7.2 y)/Iran	0.1 g/Kg/d creatine + RT	10	3	There were no definitive findings regarding the role of creatine in the antioxidant system and quality of life for older adults. However, using this supplement in addition to RT can double the strength gains achieved from RT.	9	L
19	Deacon et al. 2008 [51]	80/ male: 50 female: 30	COPD patients referred for pulmonary rehabiliation (PR) (68.2 ± 8.2 y)	Creatine (22 g/day loading; 3.76 g/day maintenance) vs. placebo + PR (aerobic and resistance exercises.)	7	3	Functional performance and strength improved similarly in both groups after PR with no additional benefits from creatine over PR alone.	7	H
20	Cooke et al. 2014 [52]	20/all male	Healthy recreationally active men (55–70 y)	Creatine monohydrate and carbohydrate (20 g/day and 5 g/day respectively for 7 days, then 0.1 g/day and 5 g/day on training days respectively) vs. placebo carbohydrate (20 g/day for 7 days, then 5 g on training days) + RT	12 weeks	3	Body composition and muscle strength improved for both groupsfrom thr resistance training with no additional benefits from the creatine supplementation.	9	S

*Abbreviations:H High risk of bias, *S* Some concerns, *L* Low risk of bias

Studies that used dietary supplements along with creatine were excluded to merely focus our analysis on the added effects of creatine. Furthermore, the vast majority of the included randomized trials (16 out of 20) either monitored participants’ dietary intake and macronutrient distribution or instructed the participants to not alter their dietary intake. Dietary monitoring was typically incorporated through 3-day food records or 24-hour dietary recalls. However, four studies (Faager et al. 2006, Hass et al. 2007, and Eijnde et al. 2003, Deacon et al. 2008) did not explicitly detail methods for monitoring or standardizing dietary intake or macronutrient distribution for the purpose of controlling study outcomes. This suggests that dietary intake was largely maintained and unlikely to introduce a confounding effect on creatine supplementation outcomes [38, 40, 44, 51]. A total of 1093 participants were included in the study, consisting of 69% females and 31% males.

### Effects of creatine supplementation and exercise training on 1RM

**Fig. 2:**
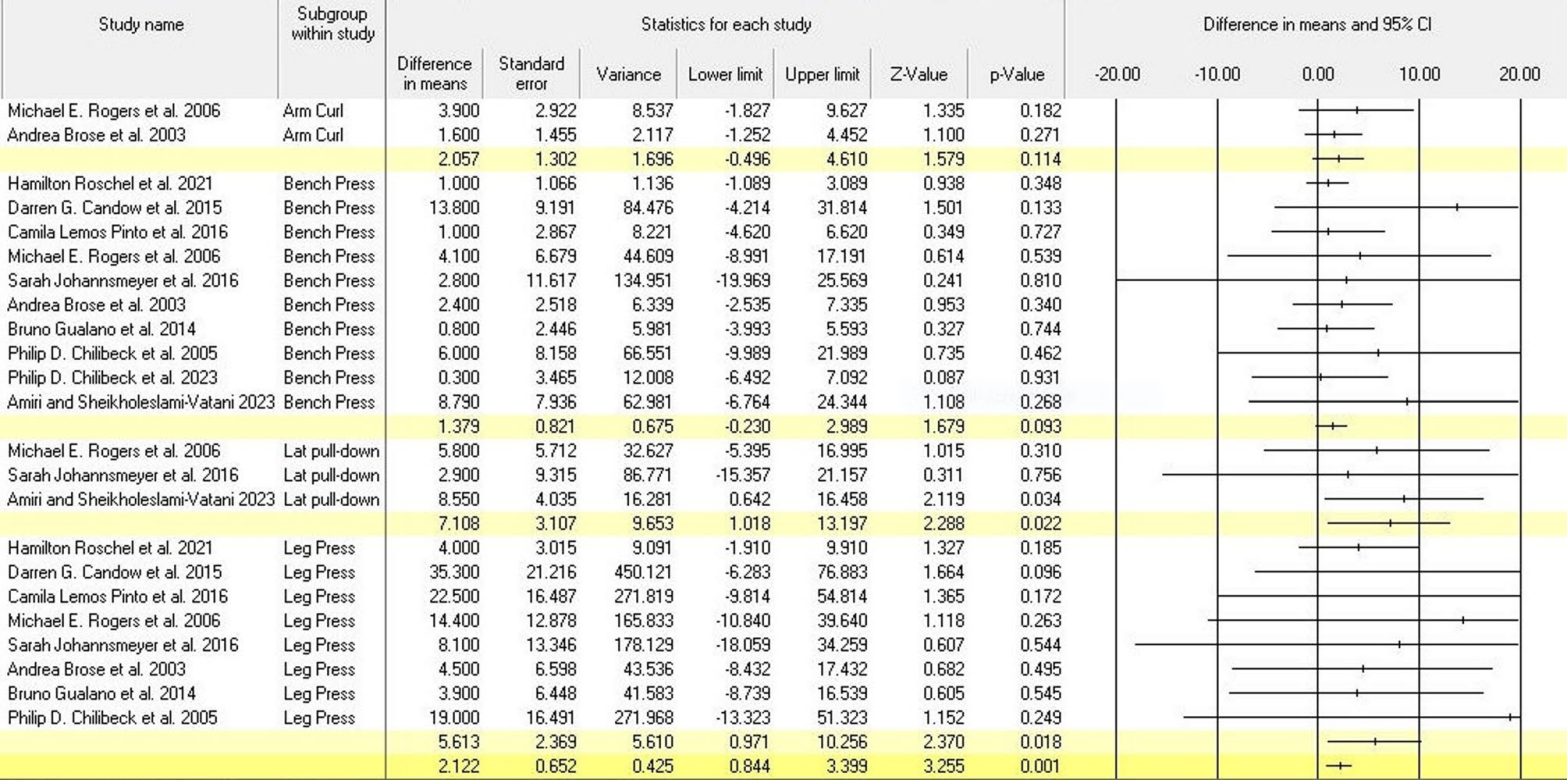
Meta-analysis of the effects of exercise training and creatine supplementation on 1RM (kg) in older adults

We employed forest plots in the meta-analysis to illustrate the I² statistics, which reflect the level of heterogeneity. The analysis revealed no significant heterogeneity between studies (I² = 0%, *P* = 0.876). Our results indicated a significant impact of creatine supplementation and exercise training on 1RM (kg) in older adults (mean difference = 2.122, Z = 3.255, *P* = 0.001; see Fig. 2). In a subgroup analysis that included Arm Curl (*P* = 0.114), Bench Press (*P* = 0.195), Chest Press (*P* = 0.181), Lat Pull-Down (*P* = 0.022), and Leg Press (*P* = 0.018), we observed significant differences in 1RM between the intervention and control groups in older adults (Arm Curl, Bench Press, and Chest Press showed no significant differences). Finally, exercise training enhances muscle strength in older adults by increasing 1RM.

### Effects of creatine supplementation and exercise training on BMD

**Fig. 3:**
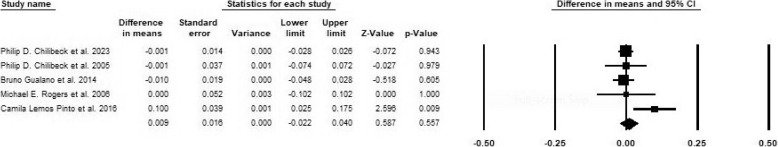
Meta-analysis of the effects of exercise training and creatine supplementation on BMD of total body (g/cm^2^) in older adults

Creatine supplementation and exercise training did not significantly affect bone mineral density (BMD) of total body (g/cm^2^) in older adults, with a mean difference of 0.009, Z = 0.587, and a P-value of 0.557 (see Fig. 3). Regarding heterogeneity and publication bias, there was no significant publication bias based on Begg’s test (*P* = 0.141) and Egger’s test (*P* = 0.412), which examine the association between the observed treatment effects and their standard errors, where a strong association implies publication bias. We used forest plots to display the I² statistics, indicating the heterogeneity level. The analysis shows no significant heterogeneity between the studies (I² = 41%, *P* = 0.142). The sensitivity analysis was assessed via one study removal method. Removing any individual study did not change the overall result, and the results of the meta-analysis remained stable without significant changes.

### Effects of creatine supplementation and exercise training on body fat

**Fig. 4:**
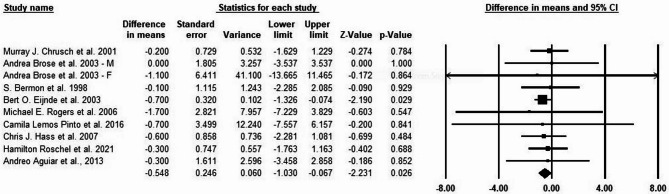
Meta-analysis of the effects of exercise training and creatine supplementation on body fat (%) in older adults

**Fig. 5:**
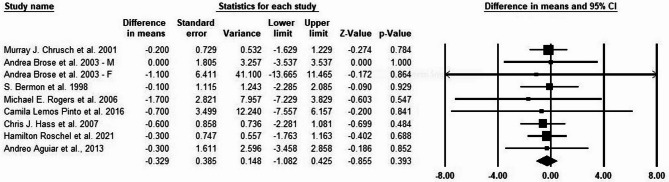
Meta-analysis of the effects of exercise training and creatine supplementation on body fat (%) in older adults after excluding the study B. O. Ejinde et al., 2003

Our results indicate a significant impact of creatine supplementation and exercise training on reducing body fat (%) in older adults (mean difference = −0.548, Z = −2.231, *P* = 0.026; see Fig. 4), which suggests that combining exercise training with creatine supplementation can improve body composition in this population. There was no significant publication bias in our assessment of heterogeneity and publication bias based on Begg’s (*P* = 0.162) and Egger’s (*P* = 0.206) tests. We used forest plots in the meta-analysis to illustrate the I² statistics, which indicate heterogeneity among studies. The analysis revealed no significant heterogeneity between studies (I² = 0%, *P* = 0.999). We also evaluated the sensitivity analysis via the one study removal method. This evaluation indicated that the overall result would change if we excluded the study by Ejinde et al. (2003), which had a high weight in the analysis. Therefore, we performed the meta-analysis again without including the study by Ejinde et al. (2003). The updated results in Fig. 5 indicated a mean difference of −0.329, Z = −0.855, and *P* = 0.393.

## Discussion

The present meta-analysis was performed on 18 randomized-control trials to analyze the effects of creatine supplementation on physical training (> 2 weeks) outcomes in older adults (≥ 55 years old). Our analysis revealed that adding creatine supplementation to exercise training significantly increases the 1RM test results and muscle strength in older adults [53–55]. Our results also correspond with the findings from previous randomized control trials on the effect of creatine supplementation during physical training on muscle strength [15, 41, 42, 45, 50]. *Amiri et al.* reported that creatine supplementation during a 10-week strength training program in older adults (mean age 68.1 ± 7.2 years old) doubles muscle strength gains compared to exercise training alone [50]. Similarly, Johannsmeyer et al. demonstrated that creatine enhances muscle strength gains in a 12-week drop-set-resistance training in aging men [42]. However, several randomized-control trials reported no added effects on muscle strength for creatine supplementation during exercise training. A 2-year randomized control trial on postmenopausal women concluded that adding creatine to strength training significantly increases lean mass tissue and walking speed but has no beneficial effect on muscle strength [39]. *Roschel et al.* also observed no significant improvement in muscle adaptations with the addition of creatine supplementation to strength training in frail and pre-frail older adults (age 72 ± 6 y) [49]. These discrepancies may be attributed to differences between frail and healthy populations’ responses to supplementation and variations in the sensitivity of different testing methods to detect small changes in muscle strength.

Our results corroborate previous meta-analysis findings on the effects of creatine on muscle strength in older adults [22, 56, 57]. According to a meta-analysis, creatine enhances the results of strength training by increasing total body mass, fat-free mass, and 1RM in the chest press and leg press, with no observed effects on fat mass [22]. In the most recent meta-analysis, Chilibeck et al. reported that creatine supplementation with strength training (2–3 sessions/week) for 7–52 weeks significantly increases lean tissue mass (about 1.37 kg) in addition to upper- and lower-body muscle strength in older adults (57–70 years old) compared to strength training alone [56]. These findings agree with our 1RM sub-group analysis results, showcasing that creatine increases 1RM in leg-press (*p* = 0.018) and lat-pulldown (*p* = 0.022) strength. Improving lower-body muscle strength with creatine during exercise training is especially vital for older adults, as these muscle groups tend to lose mass, power, and strength more markedly with age than upper-body muscles [58].

Exercise training enhances muscle strength mainly by facilitating muscle hypertrophy via the release of growth hormone [59–61], the IGF-1/Akt/mTOR pathway [62–64], satellite cell activation [61, 62], and myostatin inhibition [65, 66]. It also enhances muscle metabolism and energy production by activating Ca^2+^-dependent signaling pathways [64], AMPK signaling [64] and maintaining mitochondrial quality and quantity, which decline with muscular aging [64, 67–69]. Creatine supplementation amplifies these effects by increasing intramuscular phosphocreatine (PCr) -providing an energy buffer and higher body capacity for exercise training [56] and promoting satellite cell proliferation [70–72], IGF-1 recruitment [72, 73] and glycogen accretion [70, 74], in addition to its anti-catabolic effects [75]. These events help maintain energy levels during short-duration, high-intensity exercises and promote muscle recovery, enhancing the body’s ability to train with greater volume and improve adaptation.

Our initial analysis indicated that combined exercise and creatine supplementation significantly affect body fat percentage, aligning with findings from Aguiar et al. [47] and Brose et al. [20] regarding changes in body composition, particularly fat-free mass. The mechanisms behind these findings may involve enhancing intramuscular creatine stores, which can elevate ATP resynthesis and improve exercise performance, resulting in greater muscle hypertrophy and a higher metabolic rate, thereby reducing body fat percentage [76–79].

In our evaluation of bias risk using the one-study-removed method, we found that most studies included in this meta-analysis showed no significant change in body fat percentage following the combined use of creatine supplements and exercise in older adults. Notably, the results for body fat percentage were no longer significant after excluding the study by Eijnde et al. [44], which had a larger sample size, thereby carrying more weight in the overall analysis. Consequently, when we removed this study, our overall findings indicated no significant change in body fat percentage. Given these conflicting results, there appears to be insufficient evidence to support a definitive conclusion regarding the effects of creatine supplementation and exercise on body fat percentage in older adults. Further research with larger and more consistent sample sizes is necessary to understand these findings better and reach conclusive results.

Our meta-analysis indicates that physical activity in combination with creatine has no significant effect on BMD in older adults. This finding aligns with the results of most studies included in our analysis, such as those by Chilibeck et al. (2015) and Rogers et al. (2005) [45, 80]. The lack of significant findings in our analysis may be due to several factors, one of which is the length of the interventions. In many studies, this duration may have been too short to observe changes in BMD. Additionally, there is a lack of literature addressing this issue.

Additionally, variations in the types and intensities of the exercise protocols used across studies could have influenced the outcomes. Differences in baseline health and training intensity may explain variations in results across studies. Some studies included participants with specific health conditions, Some research included participants with specific health conditions, such as frailty or Parkinson’s disease [38, 49], which may lead to less effective responses to exercise and creatine than healthy older adults [20]. Similarly, studies involving participants with bone conditions like osteopenia or osteoporosis [15] might demonstrate different effects on BMD compared to studies with participants who do not have these conditions.

To better understand the potential effects of creatine and exercise on BMD, we suggest that future research focus on longer intervention periods and include specific weight-bearing or high-impact exercises known to stimulate bone growth. Moreover, performing more large-scale randomized controlled trials to confirm these findings would be beneficial.

Previous randomized control trials on older adults have examined various dosing regimens for creatine monohydrate. Loading a dose of 20 g/d for 5 to 7 days and 5–7 g/d has improved muscle strength during exercise training [15, 21, 38, 81]. Likewise, 5–7 g/d (or 0.1 g/kg/day) creatine monohydrate has also augmented the effects of exercise training on muscle strength and lean mass [20, 46, 47, 80]. In previous studies, no adverse kidney and liver function events have been associated with these dose regimens. However, gastrointestinal events were previously reported with both dose regimens, especially during the loading phase in older adults, which lowers patient compliance and adherence to the supplement administration [41]. Studies show that the additive effects of creatine monohydrate supplementation on muscle strength gains during exercise training are observed with treatment durations exceeding 12 weeks, and no serious adverse effects have been noted for up to two years [15, 20, 21, 38, 46, 47, 80, 81]. Further studies are required to compare the effectiveness and adverse effects of the two dosing regimens and to establish the appropriate dosing and intervals between courses of supplementation for older adults. Furthermore, additional longitudinal cohort studies are necessary to investigate the persistence of creatine supplementation effects on muscle strength after discontinuation and the adverse effects of long-term creatine use in older adults, particularly since this population exhibits decreased kidney and liver function compared to young, healthy individuals [82–84].

The results of this meta-analysis on the benefits of creatine supplementation in conjunction with exercise training show significant potential for enhancing the health and well-being of older adults. The combined effects of these strategies on muscle strength directly address sarcopenia, a common condition in aging populations characterized by the progressive loss of muscle mass and function [49, 58, 85]. Sarcopenia contributes to issues such as cachexia, osteoporosis, and frailty, which can further reduce mobility, increase the risk of falls and fractures, and deteriorate the quality of life for older adults [49, 58, 85]. Physical training, especially resistance training, is recognized as the most effective intervention for combating sarcopenia. Combined with creatine supplementation, it can further enhance muscle health in older individuals [57, 75, 86]. Our findings highlight the importance of integrating creatine supplementation into exercise training programs for older adults to improve their physical independence and overall quality of life. However, more research is necessary to establish specific dosing guidelines and assess creatine supplementation’s long-term safety in older populations.

The literature review for this meta-analysis focused on three search engines and databases: PubMed, Scopus, and Web of Science. Due to their age, certain studies’ data could not be acquired, and the original datasets are no longer accessible. Moreover, given that the included studies were of moderate quality according to the Cochrane RoB 2 tool, the results of the present study should be interpreted with caution. Furtheremore, despite heterogeneity across study populations, subgroup analyses based on sample characteristics (e.g., healthy, frail, or diseased older adults) or intervention specificities (different doses and types of exercise) were not plausible since, there will be limited number of studies (only one or two) in each potential subgroup, insufficient for a robust meta-analysis. Expanding the existing literature on this topic will allow distinct analysis of these variables across diverse populations in future studies, thereby provides more accurate results.

## Conclusion

This systematic review and meta-analysis found that older adults can enhance their functional performance and body composition by participating in exercise training programs and creatine supplementation. However, more research is needed to determine the effects of exercise training and creatine supplementation on BMD.

## Data Availability

No datasets were generated or analysed during the current study.

## Abbreviations

PRISMA: Preferred reporting items for systematic reviews and meta-analyses
PEDro: Physiotherapy evidence database
1RM: One repetition maximum
BMD: Bone mineral density
RCT: Randomized clinical trials
RT: Resistance training
PD: Parkinson disease
COPD: Chronic obstructive pulmonary disease
